# Drug use differs by care level. A cross-sectional comparison between older people living at home or in a nursing home in Oslo, Norway

**DOI:** 10.1186/s12877-019-1064-8

**Published:** 2019-02-19

**Authors:** Amura Francesca Fog, Jørund Straand, Knut Engedal, Hege Salvesen Blix

**Affiliations:** 1 Nursing Home Agency, Oslo Municipality, Oslo, Norway; 2 General Practice Research Unit, Department of General Practice, Institute of Health and Society, University of Oslo, Postbox 1130 Blinderen, N-0318 Oslo, Norway; 3 Norwegian National Advisory Unit for Aging and Health, Vestfold County Hospital HF, Toensberg and Oslo University Hospital, Oslo, Norway; 4 Department of Drug Statistics, Norwegian Public Institute of Health, Oslo, Norway

**Keywords:** Older people, Community, Nursing homes, Drug use, Prevalence, Prescription database, Dementia, Cardiovascular drugs, Opioids, Psychotropic drugs

## Abstract

**Background:**

Drug consumption increases with age, but there are few comparisons of drug use between old people living at home or in a nursing home. To identify areas of concern as well as in need for quality improvement in the two settings, we compared drug use among people aged ≥70 years living at home or in a nursing home.

**Methods:**

Cross-sectional observational study from Oslo, Norway. Information about drug use by people living at home in 2012 was retrieved from the Norwegian Prescription Database. Drug use in nursing homes was recorded within a comprehensive medication review during November 2011–February 2014. Prevalence rates and relative risk (RR) with 95% confidence intervals were compared between uses of therapeutic groups with prevalence rates of ≥5%. Drug use was compared for the total population and by gender and age group.

**Results:**

Older people (both genders) in nursing homes (*n* = 2313) were more likely than people living at home (*n* = 48,944) to use antidementia drugs (RR = 5.7), antipsychotics (RR = 4.0), paracetamol (RR = 4.0), anxiolytics (RR = 3.0), antidepressants (RR = 2.8), dopaminergic drugs (RR = 2.7), antiepileptic drugs (RR = 2.4), loop diuretics (RR = 2.3), cardiac nitrates (RR = 2.1) or opioids (RR = 2.0). By contrast, people living in a nursing home were less commonly prescribed statins (RR = 0.2), nonsteroidal antiinflammatory drugs (NSAIDs) (RR = 0.3), osteoporosis drugs (RR = 0.3), thiazide diuretics (RR = 0.4), calcium channel blockers (RR = 0.5) or renin–angiotensin inhibitors (RR = 0.5). Each of the populations had only minor differences in drug use by gender and a trend towards less drug use with increasing age (*p* <  0.01).

**Conclusions:**

Drug use by older people differs according to care level, and so do areas probably in need for quality improvement and further research. In nursing home residents, this relates to a probable overuse of psychotropic drugs and opioids. Among older people living at home, the probable overuse of NSAIDs and a possible underuse of cholinesterase inhibitors and osteoporosis drugs should be addressed.

## Background

With the aging of populations, substantially more people are living with multi-morbidity [1, 2], dementia [3] or frailty [4]. Despite the prevalence of these conditions, older people today live longer with fewer functional limitations and disabilities than equally olds in earlier generations [5]. In Norway, it is a national priority to support people living in their own homes as long as possible. Based on individual needs and regardless of income, the municipality is responsible for providing either home-based nursing services or long-term care in a nursing home.

Direct comparisons of morbidity between older persons living at home and in nursing homes are lacking, but cognitive impairment, neuropsychiatric symptoms, Parkinson’s disease and stroke are all more prevalent in nursing home residents than among those living in the community [6]. In Norway, almost half of all deaths take place in nursing homes [7].

Older people use more drugs than any other group, although the scientific evidence for drug efficacy and safety is limited, particularly for those older than 80 years. In Norway, people older than 67 years represent about 15% of the population but use 45% of all prescription drugs [8], the vast majority prescribed by general practitioners.

Because of differences in morbidity and life expectancy between older people living at home or in a nursing home, it is likely that the drug use differs by care level, for example with more symptomatic and palliative approach in the nursing home setting. It has been reported that nursing home residents use more psychotropic drugs and fewer cardiovascular drugs than their age-matched peers living in the community [9–11]. Clinical guidelines rarely take the clinical setting, or a patient’s multi-morbidity or limited life expectancy into consideration. Strict adherence to guidelines may therefore also contribute to inappropriate polypharmacy in older people [12]. Due to the differences in morbidity, disability and drug use between older people residing in nursing homes or at home, measures to improve drug prescription practice for older people needs to be tailored for the care-level setting in question. For planning of future quality improvement studies to fit with the clinical setting, it is therefore relevant to analyse both differences and similarities in drug use between older people in these two settings. This may ensure that the most important problems in each setting will be addressed. Describing drug use patterns for older people residing in the two settings may identify areas of concern as well as in need for quality improvement, and it may guide the focus for further research into the field.

In this study we have described the drug use in older people living at home or in a nursing home to identify the most pronounced differences in drug use, aiming also to identify areas of concern as well as in need for quality improvement of the drug use in the two settings.

## Methods

We collected information about the drug use of people aged 70 years old or older who were living at home or in a nursing home in the Oslo municipality, Norway from two sources.

Drug use for people living at home in 2012 (*n* = 48,944 people) was retrieved from the Norwegian Prescription Database (NorPD). The NorPD contains information about all prescription drugs dispensed at pharmacies in Norway and covers all people living in the country, except those living in long-term care institutions such as nursing homes [8].

The drug use data for people living in a nursing home were retrieved from the database of a medication review project performed at 41 of 51 nursing homes in Oslo municipality during November 2011–February 2014 [13]. The 41 nursing homes were representative of nursing homes in the municipality. From that project’s baseline data (i.e., before the medication review), we retrieved information about the drug use of the long-term nursing home residents ≥70 years old (*n* = 2313) [13].

For both populations, the collected datasets included information about the drug names and the person’s gender and age group (70–79 years, 80–89 years and ≥ 90 years).

Drugs were categorized according to the Anatomical Therapeutic Chemical (ATC) classification system [14].

### Statistical analyses

The drug use prevalence rate in the population living at home was defined as the number of people who received at least one supply of a drug in 2012. In Norway, drugs for chronic and stable use may be prescribed for 1 year’s use, however dispensed at pharmacies in quantities corresponding to about 3 months’ use. Therefore, we calculated drug prevalence rates based on purchase data for both 3 months and 12 months. Because the prevalence rates were almost identical, we have used the annual drug prevalence rates for the statistical analyses for reasons of feasibility.

The drug use prevalence rate in the nursing home population was defined as the number of people who used the drug in question at the time of the medication review. We defined the same drug issued both regularly and as needed (pro re nata, prn) to the same person living in a nursing home as one prescription. This approach fits better to the data available from the general outpatient population and it is often used in pharmaco-epidemiological studies based on registry data [15].

The drug use in the two populations was compared in STATA SE 14 (Stata Corp LP, College Station, TX) using the relative risk (RR) with 95% confidence interval (95% CI) and the population living at home as the reference group. The analyses were performed also by gender and by age group. Associations between drug use and age group were determined using the chi-squared test for trend in proportions or Cochran–Armitage test for trend [16]. The level of significance was set at 0.05. We report statistical significance with RR (95% CI). We interpreted the findings on the assumption that differences reach clinical significance when the RR was ≤0.5 or ≥ 2.

## Results

People living in a nursing home (*n* = 2313) were generally older and were more often women than those living at home (*n* = 48,944) (Table 1).

**Table 1:** Demographic characteristics of old people living at home and in a nursing home

Demographic characteristics	H (n = 48,944)	NH (*n* = 2313)
Women, n (%)	29,326 (59.9)	1752 (76.0)
Age 70–79 years, n (%)	27,299 (55.8)	311 (13.4)
Age 80–89 years, n (%)	17,645 (36.0)	1023 (44.3)
Age 90+ years, n (%)	4000 (8.2)	979 (42.3)

H (home population), NH (nursing home population). Gender information was missing for seven NH residents

Compared with people living at home (the reference group), people living in a nursing home more frequently used antidementia drugs [RR = 5.7)], antipsychotics (RR = 4.0), paracetamol (RR = 4.0), anxiolytics (RR = 3.0), antidepressants (RR = 2.8), dopaminergic drugs (RR = 2.7), antiepileptic drugs (RR = 2.4), loop diuretics (RR = 2.3), cardiac nitrates (RR = 2.1) or opioids (RR = 2.0) (Table 2).

**Table 2:** Drug use in older people living in a nursing home versus those living at home

Drug therapeutic group (ATC-3th level)^a^	Drug use prevalence (%)		Relative Risk (RR)	
	H (n = 48,944)	NH (*n* = 2313)	95% CI	*p*-value
Opioids	22.7	46.3	2.0 (2.0, 2.1)	<0.01
Paracetamol	18.2	72.1	4.0 (3.9, 4.1)	<0.01
Antiepileptic drugs	4.0	9.5	2.4 (2.1, 2.7)	<0.01
Dopaminergic drugs	1.5	3.9	2.7 (2.2, 3.4)	<0.01
Antipsychotics	4.0	17.2	4.3 (3.9, 4.8)	<0.01
Anxiolytics	16.2	48.4	3.0 (2.9, 3.1)	<0.01
Hypnotics/sedatives	28.6	49.2	1.7 (1.7, 1.8)	<0.01
Antidepressants	11.2	31.6	2.8 (2.7, 1.8)	<0.01
Antidementia drugs	2.0	11.4	5.7 (5.0, 6.5)	<0.01
Cardiac glycosides	3.2	5.8	1.8 (1.5, 2.2)	<0.01
Cardiac nitrates	7.5	15.4	2.1 (1.9, 2.3)	<0.01
Loop diuretics	14.0	31.7	2.3 (2.1, 2.4)	<0.01
Other diuretics^b^	6.6	2.9	0.4 (0.3, 0.6)	<0.01
Beta-blockers	29.1	24.8	0.9 (0.8, 0.9)	<0.01
Calcium channel blockers	19.6	9.0	0.5 (0.4, 0.5)	<0.01
Renin–angiotensin drugs	41.1	18.6	0.5 (0.4, 0.5)	<0.01
Statins	35.0	5.7	0.2 (0.1, 0.2)	<0.01
Antithrombotic agents^c^	47.4	44.0	0.9 (0.9, 1.0)	<0.01
Inhalators^d^	17.8	18.3	1.0 (0.9, 1.1)	0.55
Corticosteroids, systemic	10.2	6.6	0.7 (0.6, 0.8)	<0.01
Antihistamines, systemic	11.6	8.2	0.7 (0.6, 0.8)	<0.01
Vitamin B_12_ and folic acid	6.5	13.6	2.1 (1.9, 2.4)	<0.01
Thyroid therapy	10.9	16.1	1.5 (1.4, 1.6)	<0.01
Peptic ulcer drugs^e^	17.2	21.4	1.3 (1.2, 1.4)	<0.01
Antidiabetics^f^	10.2	11.1	1.1 (1.0, 1.2)	0.15
Drugs for glaucoma	9.3	10.4	1.1 (1.0, 1.3)	0.08
Oestrogens	6.3	4.6	0.7 (0.6, 0.9)	<0.01
Benign prostate hypertrophy drugs	6.1	2.3	0.4 (0.3, 0.5)	<0.01
Osteoporosis drugs	6.5	2.1	0.3 (0.3, 0.4)	<0.01
NSAIDs	20.7	5.5	0.3 (0.2, 0.3)	<0.01

H (home population), NH (nursing home population). RR (relative risk with 95% confidence interval and p-value and the population living at home as the reference group)

^a^Drugs with prevalence rates ≥5% in at least one of the populations, except for dopaminergic drugs

^b^The vast majority were thiazides

^c^Mostly anti-platelet agents, such acetylsalicylic acid (36.2% vs. 31.2%) and warfarin (11.5% vs. 9.0%)

^d^Steroid, adrenergic and anticholinergic inhalators

^e^The vast majority were proton pump inhibitors

^f^Oral antidiabetic drugs RR = 2.4(2.0, 2.9) and insulin RR = 0.7(0.6, 0.9)

By contrast, people living in a nursing home were less commonly issued statins (RR = 0.2), nonsteroidal antiinflammatory drugs (NSAIDs) (RR = 0.3), osteoporosis drugs (RR = 0.3), thiazide diuretics (RR = 0.4), calcium channel blockers (RR = 0.5) or renin–angiotensin drugs (RR = 0.5) (Table 2).

The RRs for use of opioids, antipsychotics, anxiolytics, hypnotic/sedatives, antidepressants and antidementia drugs in nursing home residents compared with those living at home are presented in Table 3. In particular clomethiazole (RR 68.8), haloperidol (RR = 15.8), memantine (RR = 15.0), risperidone (RR = 14.0), morphine (RR = 13.7), fentanyl (RR = 12.4) and buprenorphine (RR = 10.7) were indeed more often issued for nursing home residents than for older people living at home. Weak opioids were frequently used in both settings; the use of tramadol was higher in nursing homes (RR = 1.8), but the use of codeine-containing analgesics did not differ between populations (RR = 1.0).

**Table 3:** Use of particular drugs in older people living in a nursing home or at home

Drugs (ATC-5th level)^a^	Drug use prevalence (%)		Relative Risk (RR)	
	H (*n* = 48,944)	NH (*n* = 2313)	95% CI	*p*-value
Morphine	0.3	4.5	13.7 (10.7, 17.4)	<0.01
Oxycodone	1.8	10.1	5.7 (4.9, 6.5)	<0.01
Fentanyl	0.5	6.2	12.4 (1.1, 15.2)	<0.01
Buprenorphine	1.1	11.9	10.7 (9.3, 12.3)	<0.01
Codeine analgesics	17.3	17.5	1.0 (0.9, 1.1)	0.75
Tramadol	6.8	12.5	1.8 (1.6, 2.1)	<0.01
Haloperidol	0.3	4.1	15.8 (12.1, 20.6)	<0.01
Clozapine, olanzapine, quetiapine	0.8	5.3	6.9 (5.6, 8.4)	<0.01
Risperidone	0.4	5.4	14.0 (11.2, 17.5)	<0.01
Oxazepam	7.7	41.7	5.5 (5.2, 5.8)	<0.01
Diazepam	8.1	8.6	1.1 (0.9, 1.2)	0.35
Zopiclone, zolpidem	27.3	39.8	1.5 (1.4, 1.5)	<0.01
Clomethiazole	0.1	8.5	68.8 (51.8, 91.4)	<0.01
Citalopram, escitalopram sertraline, paroxetine	6.4	19.7	3.1 (2.9, 3.4)	<0.01
Mianserin, mirtazapine, venlafaxine	4.6	14.8	3.2 (2.9 3.6)	<0.01
Donepezil, rivastigmine, galantamine	1.8	5.7	3.2 (2.7, 3.9)	<0.01
Memantine	0.4	5.7	15.0 (12.1, 18.7)	<0.01

H (home population), NH (nursing home population). RR (relative risk with 95% confidence interval and p-value and the population living at home as the reference group)

^a^Drugs with prevalence rates ≥4% in at least one of the populations

Differences in drug use by gender are presented in Table 4. Except for opioids, the differences between home and nursing home populations in the use of drugs affecting the nervous system were larger for men than for women (home population as the reference group).

**Table 4:** Drug use in women and men living in a nursing home or at home

Drug groups	Women (*n* = 31,078)			Men (*n* = 20,152)			Women vs men	
	H	NH		H	NH		H	NH
	%	%	RR^a^ (95% CI)	%	%	RR^a^ (95% CI)	RR^b^ (95% CI)	RR^b^ (95% CI)
Opioids	25.7	48.6	2.3 (2.2, 2.4)	18.3	38.7	2.2 (2.0, 2.5)	1.2 (1.2, 1.2)	1.1 (1.1, 1.2)
Paracetamol	22.4	73.6	3.3 (3.2, 3.4)	12.0	67.7	5.9 (5.5, 6.3)	1.3 (1.3, 1.3)	1.1 (1.0, 1.2)
Antiepileptic drugs	4.1	8.5	2.1 (1.8, 2.5)	3.9	12.6	3.4 (2.7, 4.3)	1.0 (1.0, 1.1)	0.9 (0.8, 1.0)
Dopaminergic agents	1.2	3.2	2.6 (2.0, 3.4)	1.8	6.3	3.7 (2.7, 5.2)	0.8 (0.8, 1.0)	0.8 (0.7, 1.0)
Antipsychotics	4.7	17.1	3.7 (3.3, 4.1)	2.9	17.5	6.2 (5.1, 7.6)	1.2 (1.1, 1.2)	1.0 (0.9, 1.1)
Anxiolytics	20.1	49.2	2.5 (2.3, 2.6)	10.5	45.9	4.6 (4.1, 5.0)	1.3 (1.2, 1.3)	1.0 (1.0, 1.1)
Hypnotics/sedatives	34.3	49.1	1.4 (1.4, 1.5)	20.2	49.5	2.6 (2.3, 2.8)	1.3 (1.2, 1.3)	1.0 (1.0, 1.1)
Antidepressants	13.8	33.0	2.4 (2.2, 2.6)	7.3	27.7	3.9 (3.4, 4.5)	1.3 (1.2, 1.3)	1.1 (1.0, 1.1)
Antidementia drugs	2.2	10.8	5.0 (4.3, 5.8)	1.8	13.7	8.0 (6.4, 0.1)	1.1 (1.0, 1.1)	0.9 (0.9, 1.0)
Cardiac glycosides	3.2	5.7	1.8 (1.5, 2.2)	3.2	5.9	1.9 (1.4, 2.7)	1.0 (1.0, 1.0)	1.0 (0.9, 1.1)
Cardiac nitrates	7.1	15.8	2.2 (2.0, 2.5)	8.2	14.1	1.8 (1.5, 2.2)	0.9 (0.9, 1.0)	1.0 (1.0, 1.1)
Loop diuretics	14.7	32.2	2.2 (2.0, 2.4)	12.9	29.9	2.4 (2.1, 2.8)	1.1 (1.0, 1.1)	1.0 (1.0, 1.1)
Other diuretics	7.5	3.0	0.4 (0.3, 0.5)	5.3	2.3	0.5 (0.3, 0.8)	1.2 (1.1, 1.2)	1.1 (0.9, 1.2)
Beta-blockers	27.1	25.1	0.9 (0.9, 1.0)	32.1	23.6	0.8 (0.7, 0.9)	0.9 (0.9, 0.9)	1.0 (1.0, 1.1)
Calcium channel blockers	19.2	9.5	0.5 (0.4, 0.6)	20.1	7.6	0.4 (0.3, 0.5)	1.0 (0.9, 1.0)	1.1 (1.0, 1.1)
Renin–angiotensin	40.1	17.9	0.4 (0.4, 0.5)	42.6	20.7	0.5 (0.4, 0.6)	1.0 (0.9, 1.0)	1.0 (0.9, 1.0)
Statins	31.8	4.7	0.1 (0.1, 0.2)	39.7	9.2	0.2 (0.2, 0.3)	0.9 (0.8, 0.9)	0.8 (0.7, 0.9)
Antithrombotic agents	42.7	42.0	1.0 (0.9, 1.0)	54.6	50.1	1.0 (0.9, 1.0)	0.8 (0.8, 0.8)	0.9 (0.9, 1.0)
Inhalators	18.6	17.8	1.0 (1.0, 1.1)	16.5	23.1	1.0 (1.0, 1.0)	1.1 (1.0, 1.1)	0.9 (0.9, 1.0)
Corticosteroids	11.1	6.3	0.6 (0.5, 0.7)	8.9	7.4	0.9 (0.6, 1.2)	1.1 (1.1, 1.1)	1.0 (0.9, 1.1)
Antihistamines	13.6	7.6	0.6 (0.5, 0.7)	8.7	9.9	1.2 (0.9, 1.5)	1.2 (0.2, 1.2)	0.9 (0.8, 1.0)
Drugs for peptic ulcer	18.2	21.7	1.2 (1.1, 1.3)	15.7	20.0	1.3 (1.1, 1.6)	1.1 (1.1, 1.1)	1.0 (1.0, 1.1)
Antidiabetics	8.5	8.6	1.0 (0.9, 1.2)	12.8	11.7	0.9 (0.7, 1.2)	0.8 (0.8, 0.8)	0.9 (0.8, 1.0)
Osteoporosis drugs	9.8	2.6	0.3 (0.2, 0.4)	1.4	0.7	0.5 (0.2, 1.4)	1.6 (1.6, 1.0)	1.2 (1.1, 1.3)
NSAIDs	22.7	5.6	0.6 (0.5, 0.7)	17.8	5.2	0.3 (0.2, 0.4)	1.1 (1.0, 1.1)	1.0 (0.9, 1.1)

H (home population), NH (nursing home population). RR (relative risk with 95% confidence interval)

Number of women (H = 29,326, NH = 1752) and men (H = 19,618, NH = 554)

^a^RR values calculated using the home population as the reference group

^b^RR values calculated using women as the reference group

Differences in drug use by age group are presented in Table 5. With the exception of antiepileptic drugs and dopaminergic agents, a negative trend in the percentage of people using particular drugs with increasing age was observed for people living at home (*p* <  0.01). For people living in a nursing home, a negative trend in the percentage of people using the drug with increasing age (*p* <  0.01) was observed for paracetamol, antiepileptic drugs, dopaminergic agents, antipsychotics, anxiolytics, antidepressants, antidementia drugs, cardiac glycoside and nitrates, loop diuretics, statins and antidiabetics (Table 5).

**Table 5:** Drug use in people living in a nursing home or at home by age groups

Drug groups	Age 70–79 years *n* = 27,610			Age 80–89 years *n* = 18,668			Age ≥ 90 years *n* = 4979			Chi-square test for trend in proportion	
	H	NH		H	NH		H	NH		*p*-value^a^	*p*-value^b^
	%	%	RR (95% CI)	%	%	RR (95% CI)	%	%	RR (95% CI)		
Opioids	20.4	51.0	2.5 (2.2, 2.8)	25.0	42.8	1.7 (1.6, 1.8)	28.4	48.5	1.7 (1.6, 1.9)	< 0.01	0.71
Paracetamol	14.0	68.3	4.9 (4.5, 5.3)	21.7	70.0	3.2 (3.1, 3.4)	31.6	75.5	2.4 (2.3, 2.6)	< 0.01	< 0.01
Antiepileptic drugs	3.9	20.8	5.4 (4.3, 6.8)	4.2	9.4	2.2 (1.8, 2.7)	3.5	6.0	1.7 (1.3, 2.4)	0.84	< 0.01
Dopaminergic agents	1.4	7.4	5.3 (3.5, 7.9)	1.6	4.7	2.9 (2.1, 3.8)	1.0	2.0	2.2 (1.3, 3.7)	0.48	< 0.01
Antipsychotics	3.8	25.0	6.7 (5.5, 8.2)	4.3	18.4	4.3 (3.7, 5.0)	4.4	13.5	3.1 (2.5, 3.8)	< 0.01	< 0.01
Anxiolytics	14.7	54.5	3.7 (3.3, 4.1)	17.8	49.6	2.8 (2.6, 3.0)	19.8	45.2	1.8 (1.6, 1.9)	< 0.01	< 0.01
Hypnotics/sedatives	24.0	48.1	2.0 (1.8, 2.3)	33.1	49.7	1.5 (1.4, 1.6)	41.0	49.0	1.2 (1.1, 1.3)	< 0.01	0.95
Antidepressants	10.1	39.7	4.0 (3.4, 4.6)	12.5	33.8	2.7 (2.5, 3.0)	13.3	26.8	2.0 (1.8, 2.3)	< 0.01	< 0.01
Antidementia drugs	1.1	13.5	12.4 (9.2, 16.8)	3.1	15.3	16.1 (14.1, 18.5)	3.4	6.7	2.0 (1.5, 2.6)	< 0.01	< 0.01
Cardiac glycosides	1.8	2.9	1.6 (0.9, 3.1)	4.6	5.0	1.1 (0.8, 1.4)	7.1	7.7	1.1 (0.8, 1.4)	< 0.01	< 0.01
Cardiac nitrates	5.1	9.9	1.0 (1.0, 1.0)	9.7	12.9	1.0 (1.0, 1.0)	14.6	19.8	0.9 (0.9, 1.0)	< 0.01	< 0.01
Loop diuretics	8.3	25.3	3.1 (2.5, 3.7)	18.4	27.9	1.5 (1.4, 1.7)	33.1	37.7	1.1 (1.0, 1.3)	< 0.01	< 0.01
Other diuretics	5.7	2.2	0.4 (0.2, 0.8)	7.8	2.4	0.3 (0.2, 0.5)	8.4	3.6	0.4 (0.3, 0.6)	< 0.01	0.12
Beta-blockers	25.3	21.2	0.8 (0.7, 1.0)	33.2	25.0	0.8 (0.7, 0.8)	37.6	25.8	0.7 (0.6, 0.8)	< 0.01	0.14
Calcium channel blockers	17.6	6.4	0.4 (0.2, 0.6)	21.9	9.9	0.5 (0.4, 0.5)	23.1	9.0	0.4 (0.3, 0.5)	< 0.01	0.42
Renin–angiotensin	40.1	20.8	0.5 (0.4, 0.6)	42.7	19.3	0.5 (0.4, 0.5)	40.6	17.0	0.4 (0.4, 0.5)	< 0.01	0.09
Statins	37.0	14.4	0.4 (0.3, 0.5)	35.2	5.9	0.2 (0.1, 0.2)	20.0	2.9	0.1 (0.1, 0.2)	< 0.01	< 0.01
Antithrombotic agents	41.7	39.4	0.9 (0.8, 1.1)	53.9	45.9	0.9 (0.8, 0.9)	58.5	43.5	0.7 (0.7, 0.8)	< 0.01	0.60
Inhalators	18.5	25.1	1.0 (1.0, 1.0)	17.9	20.8	1.0 (1.0, 1.0)	11.6	15.0	0.9 (0.9, 1.0)	< 0.01	< 0.01
Corticosteroids^3^	9.6	6.4	0.7 (0.4, 1.0)	11.4	8.1	0.7 (0.6, 0.9)	9.4	5.1	0.5 (0.4, 0.7)	< 0.01	0.08
Antihistamines^3^	12.7	9.3	0.7 (0.5, 1.0)	10.7	8.5	0.8 (0.6, 1.0)	8.8	7.6	0.9 (0.7, 1.1)	< 0.01	0.28
Drugs for peptic ulcer	15.8	24.7	1.6 (1.3, 1.9)	19.0	20.8	1.1 (1.0, 1.2)	18.7	20.9	1.1 (1.0, 1.3)	< 0.01	0.27
Antidiabetics	11.4	16.3	1.4 (1.1, 1.8)	9.2	10.3	1.1 (0.9, 1.4)	5.9	6.0	1.0 (0.8, 1.3)	< 0.01	< 0.01
Osteoporosis drugs	5.1	0.3	0.1 (0.0, 0.4)	8.2	3.0	0.4 (0.3, 0.5)	7.8	1.7	0.2 (0.1, 0.4)	< 0.01	0.70
NSAIDs	23.6	6.1	0.3 (0.2, 0.4)	18.1	5.2	0.3 (0.2, 0.4)	12.7	5.6	0.4 (0.3, 0.6)	< 0.01	0.93

H (home population), NH (nursing home population). RR (relative risk with 95% confidence interval and the population living at home as the reference group)

Number of people in each age group: 70–79 years (NH = 311; H = 27, 299), 80–89 years (NH = 1023; H = 17, 645) and ≥ 90 years (NH = 979; H = 4, 000)

^a^Chi-squared test for trend in proportion of drug usage at home

^b^Chi-squared test for trend in proportion of drug usage in nursing homes

## Discussion

In this comprehensive comparison of the drug use among older people living at home or in nursing homes, we have reported data on drug groups and individual drugs not commonly reported by others [9–11, 17–19]. We found large differences in drug use by people aged 70 years and older according to their place of residence.

Antidementia drugs were substantially more often used in those living in a nursing home, regardless of age or gender. This might be expected because about 80% of the nursing home residents are cognitively impaired [20], as compared with 18% of people aged 80 years and 41% of those older than 90 years living in the community [6]. However, the higher use of antidementia drugs by men than women in nursing homes (13.7% vs. 10.1%) should be investigated in further research. The use of cholinesterase inhibitors at home (1.8%) was lower than expected because these drugs are recommended palliative treatment for people with mild to moderate dementia [21, 22], most of whom are living at home. The very low use of memantine among those living at home is consistent with that the vast majority of people with severe dementia are being cared for in nursing homes.

Our observation that antipsychotics, antidepressants and anxiolytics were used more often in nursing homes is consistent with the findings of others [9–11, 18]. This probably reflects the high prevalence of significant behavioural and psychiatric symptoms of dementia (BPSD) [20] and obstacles for implementing non-pharmaceutical measures in the nursing home setting. The use of antipsychotics in nursing homes in our study was lower than in other studies [9, 11, 18, 19, 23, 24], perhaps explained by recent warnings against long term antipsychotic use because of severe side effects such as cognitive decline [25, 26], falls [27], stroke [28] and even death [29], as well as poor long-term effect on agitation [30]. We however still consider the overall use of psychotropic drugs in nursing homes to be too high because their efficacy in people with dementia is generally poor [31] and commonly harmful, and deprescribing is generally well tolerated [32, 33]. The prevalent use of clomethiazole in nursing home residents (8.5%) is surprising and it should be investigated further, given that this is a drug with a poor safety record that in general should be avoided in older people [34].

Overall, there is generally poor evidence for the efficacy of antidepressants, including selective serotonin reuptake inhibitors, in people with dementia and BPSD [35]. That the use of antidepressants in nursing homes declined with residents’ age has also been reported by others [18, 19]. A possible explanation for this may also be decreasing prevalence of depressive symptoms with increasing dementia severity [36].

Consistent with findings by others [10, 11, 19], more paracetamol and opioids were used by nursing home residents. This may partly reflect the lower use of NSAIDs for osteoarthritis in nursing home residents, but may also reflect empiric analgesic treatment in people with dementia of agitated behaviour presumed to be caused by pain [37]. That at least 20% of older people living at home use NSAIDs is a matter of concern, especially because their use might be even higher due to their possible purchase without prescription. NSAIDs are probably most commonly issued for degenerative pain without inflammation (a simple analgesic might thus be a safer option) and they pose an increased risk for gastrointestinal bleedings and adverse cardiovascular events [38]. That we in both settings found a more prevalent use of opioids than reported by others [10, 11, 18, 19] also warrants further investigation. Opioids used as part of end-of-life palliative treatment in nursing homes cannot explain this finding because palliative units were not included in the medication review.

The differences found for the uses of cardiovascular drugs between the two settings suggest that cardiovascular treatment may be more symptomatic and palliative in nursing homes than for their home-dwelling peers [10, 19, 39]. Because of the lower rate of disability and longer life expectancy among home-dwelling older people relative to nursing home residents, the potential benefits for both primary and secondary cardiovascular prevention are larger for those living at home.

Deprescribing of prophylactic drug treatment in nursing home residents with short life expectancies may explain the lower use of both statins and osteoporosis drugs by nursing home residents. However, the use of osteoporosis drugs was lower than expected among home-dwelling women because osteoporosis, with its consequent risk of fractures, is a leading health hazard in old people in Norway [40].

Based on the study results and our own clinical experience, we have identified ten drugs in particular need for critical rethinking during future educational interventions or medication reviews in the two settings (Table 6).

**Table 6:** Older people residing in nursing homes or at home: ten drugs in particular need for critical rethinking during educational interventions or medication reviews

Drug	Nursing home	Home
Antidementia drugs	Severe dementia: overuse?	Mild dementia: underuse?
Antipsychotic drugs	BPSD^1^: Too much, too long? Deprescribing should be tried	(little use)
Antidepressants	Overuse: Poor effect in people with dementia, consider tapering down dosage and deprescribing	Possible overuse: Consider tapering down dosage and deprescribing
Anxiolytics Hypnotics/sedatives	Overuse	Probable overuse
Opioids	Overuse	Probable overuse
Clomethiazole	Overuse: should be avoided whenever possible for reasons of safety	(almost no use at all)
NSAIDs	(little use)	Overuse – try paracetamol instead
Osteoporosis drugs	Possible underuse?	Possible underuse?
Statins	(little use)	Possible overuse (oldest age group)?
Drugs for peptic ulcer	Possible overuse	Possible overuse

^1^Behavioral and psychiatric symptoms in dementia

Our study has some limitations. We compared the drug use of two populations that differ in terms of morbidity and frailty without recording clinical data (e.g. diagnoses, Charleston morbidity scores, in-home care service use). We assumed that institutionalization is a proxy for frailty and a high prevalence of dementia, and we have not differentiated between robust and frail older people living at home. Drug use was investigated in terms of the prevalence of use, but we did not have access to the prescribed daily dosages or how often drugs intended for prn actually were used. Despite these limitations, we consider that our data are representative for each of the populations and have acceptable validity for identifying the most significant differences in drug use patterns between older people residing in the two settings. The prevalence rates of drug use for those living at home differed only marginally between data captured over 3 or 12 months, thus being comparable with the point prevalence data for those living in nursing homes. We included drug use data from more than half of the nursing home population in Oslo and the entire population living at home in the municipality. The patient demographics and findings in our study are also consistent with our clinical experience and with data reported in other studies [9–11, 18, 19]. We believe that the large number of participating nursing homes and the large size of the home-dwelling population account for the external validity of our findings.

## Conclusions

This study substantiates that older people living in a nursing home and at home represent two different pharmacologically realities. Further research should investigate when the changes in drug prescription occur in the process of institutionalization or if the two settings may have different therapeutic cultures that partly may explain their different prescription practices. In Norway, the inclusion of prescription data from nursing homes in the NorPD would enable monitoring their drug use over time and follow up on changes in drug use patterns.

## Abbreviations

ATC: Anatomical Therapeutic Chemical classification system
BPSD: behavioural and psychiatric symptoms of dementia
NorPD: Norwegian Prescription Database (NorPD)
NSAIDs: nonsteroidal antiinflammatory drugs
RR: relative risk with 95% confidence interval
